# Genome-wide association mapping reveals new *loci* associated with light-colored seed coat at harvest and slow darkening in carioca beans

**DOI:** 10.1186/s12870-021-03122-2

**Published:** 2021-07-20

**Authors:** Caléo Panhoca de Almeida, Isabella Laporte Santos, Jean Fausto de Carvalho Paulino, Caio Cesar Ferrari Barbosa, Cássia Cristina Augusto Pereira, Cassia Regina Limonta Carvalho, Gabriel de Moraes Cunha Gonçalves, Qijian Song, Sérgio Augusto Morais Carbonell, Alisson Fernando Chiorato, Luciana Lasry Benchimol-Reis

**Affiliations:** 1 Common Bean Genetic Group, Natural Center of Plant Genetics, Agronomic Institute (IAC), Campinas, SP Brazil; 2 Common Bean Breeding Group, Grain and Fiber Center, Agronomic Institute (IAC), Campinas, SP Brazil; 3 USDA-ARSSoybean Genomics and Improvement Lab, Beltsville, MD USA

**Keywords:** *Phaseolus vulgaris* L., Late seed coat darkening, Seed coat lightness, CIELAB scale

## Abstract

**Background:**

Common bean (*Phaseolus vulgaris* L.) is a legume whose grain can be stored for months, a common practice among Brazilian growers. Over time, seed coats become darker and harder to cook, traits that are undesirable to consumers, who associate darker-colored beans with greater age. Like commercial pinto and cranberry bean varieties, carioca beans that have darker seeds at harvest time and after storage are subject to decreased market values.

**Results:**

The goal of our study was to identify the genetic control associated with lightness of seed coat color at harvest (HL) and with tolerance to post-harvest seed coat darkening (PHD) by a genome-wide association study. For that purpose, a carioca diversity panel previously validated for association mapping studies was used with 138 genotypes and 1,516 high-quality SNPs. The panel was evaluated in two environments using a colorimeter and the CIELAB scale. Shelf storage for 30 days had the most expressive results and the L* (luminosity) parameter led to the greatest discrimination of genotypes. Three QTL were identified for HL, two on chromosome Pv04 and one on Pv10. Regarding PHD, results showed that genetic control differs for L* after 30 days and for the ΔL* (final L*—initial L*); only ΔL* was able to properly express the PHD trait. Four phenotypic classes were proposed, and five QTL were identified through six significant SNPs.

**Conclusions:**

Lightness of seed coat color at harvest showed an oligogenic inheritance corroborated by moderate broad-sense heritability and high genotypic correlation among the experiments. Only three QTL were significant for this trait – two were mapped on Pv04 and one on Pv10. Considering the ΔL, six QTL were mapped on four different chromosomes for PHD. The same HL QTL at the beginning of Pv10 was also associated with ΔL* and could be used as a tool in marker-assisted selection. Several candidate genes were identified and may be useful to accelerate the genetic breeding process.

**Supplementary Information:**

The online version contains supplementary material available at 10.1186/s12870-021-03122-2.

## Background

Common bean (*Phaseolus vulgaris* L.) is considered the most important species for production of edible dry seeds for direct consumption in the human diet [1, 2]. The nutritional value intrinsic to the grain and its potential health benefits explain the nutraceutical relevance of this legume as a source of carbohydrates, fibers, vitamins, and minerals [3], as well as of polyphenolic compounds with antioxidant properties [4]. In some African and American countries, beans provide an average of 15% of total daily calories and 36% of the protein consumed [5].

Global production of dry beans has increased 77.8% since 2012 with a record 31.5 million tons in 2017 [6]. Population studies indicate large growth in world population, and bean consumption is expected to continue to increase significantly in coming years [2]. World dry bean production is concentrated mainly in the countries of Asia and the Americas, together accounting for approximately 75% of all dry beans produced in the world [6]. Brazil is currently the third largest producer of this grain, responsible for around 75% of the production of the MERCOSUR countries and 10.4% of world production. In addition, Brazil is considered the largest consumer of this food, with average consumption of 18 kg year^−1^ per person [7].

In most countries, demand for a specific type of grain varies according to regional cultural aspects. In Brazil, carioca variety beans, characterized by a cream-colored seed coat with brown stripes [8], belonging to the Mesoamerican gene pool [9], represent up to 70% of the type of beans consumed [10, 11]. As reported for the pinto and cranberry varieties [12, 13], PHD is considered the trait with the greatest potential for devaluating the product after harvest [14, 15]. Brazilian ‘Carioca’ cultivars with dark seed coats at the time of harvest have lower added value than those with lighter-colored seed coats, as consumers associate darker seed coats with longer cooking time and old beans [8, 16, 17]. However, this common notion is incorrect as darker grains do not always indicate longer cooking time and/or older beans [15, 18].

The seed coat color and patterning traits are controlled by a complex genetic network [19]. Common bean has a wide variety of colors, and control of genetic inheritance is difficult, due to the occurrence of epistatic interactions, pleiotropic effects, multiple allelism, and linked genes [20]. PHD is commonly classified into three phenotypic classes: (1) non-darkening (ND), (2) slow darkening (SD), and (3) regular darkening (RD). Junk-Knievel et al. [21] found that a single gene controlled whether a genotype was SD or RD in pinto beans, with dominance of RD. As the SD trait is expressed in the seed coat, which is a maternal tissue, the maternal effect should be taken into account, depending on the generation that is being phenotyped [13]. According to Elsadr et al. [22], the *J* gene determines whether the grain will darken (*J*) or not (*jj*), and the *sd* gene predicts how quickly the darkening will occur (recessive epistasis). Erfatpour et al. [12] mapped the *sd* and *nd loci* on chromosome Pv07 and Pv10 in cranberry beans, respectively.

Early grain darkening interacts with several genetic, environmental, and post-harvest factors, and darkening may intensify due to humidity conditions, the drying time of the grain, and especially storage conditions [15, 23, 24]. PHD is attributed to proanthocyanidin accumulation and its subsequent oxidation in the seed coat [25, 26]. These compounds oxidize to the form of reactive quinones, which are deposited in this cellular environment, resulting in darkening of this layer [25, 27]. They are found in higher concentrations in beans with normal darkening than in those with slow darkening [27].

Regarding the carioca variety, Silva et al. [16] proposed a scale to evaluate seed darkening and suggested that control of PHD seems to be monogenic. Silva et al. [10] found that the trait is strongly affected by the genotype × environment interaction. The authors also suggested that the trait is under oligogenic control; however, the inconsistency in the segregation patterns observed shows a greater degree of complexity. Alvares et al. [28] confirmed the genotype × environment interaction and found that the genotypes evaluated did not exhibit a coinciding response in the various environments tested. Couto et al. [29] identified three microsatellite markers linked to PHD QTL in a segregating carioca population. Alvares et al. [23] identified the previously mapped Pvsd-1158 microsatellite marker linked to the *sd locus* in pinto beans [13], closely linked to the *locus* that controls slow darkening in carioca bean. However, no mapping study with markers covering the entire genome of the species included effective evaluation of PHD in the carioca bean variety or reported the lightness of color of the grain at harvest time.

Therefore, the goal of the current study was to identify carioca genomic regions associated with tolerance to PHD and HL using a genome wide association approach. For that purpose, a carioca diversity panel (CDP), previously validated for GWAS, was phenotypically characterized in two environments and genotyped using high-throughput genotyping technology.

## Results

### Phenotypic data

**Fig. 1:**
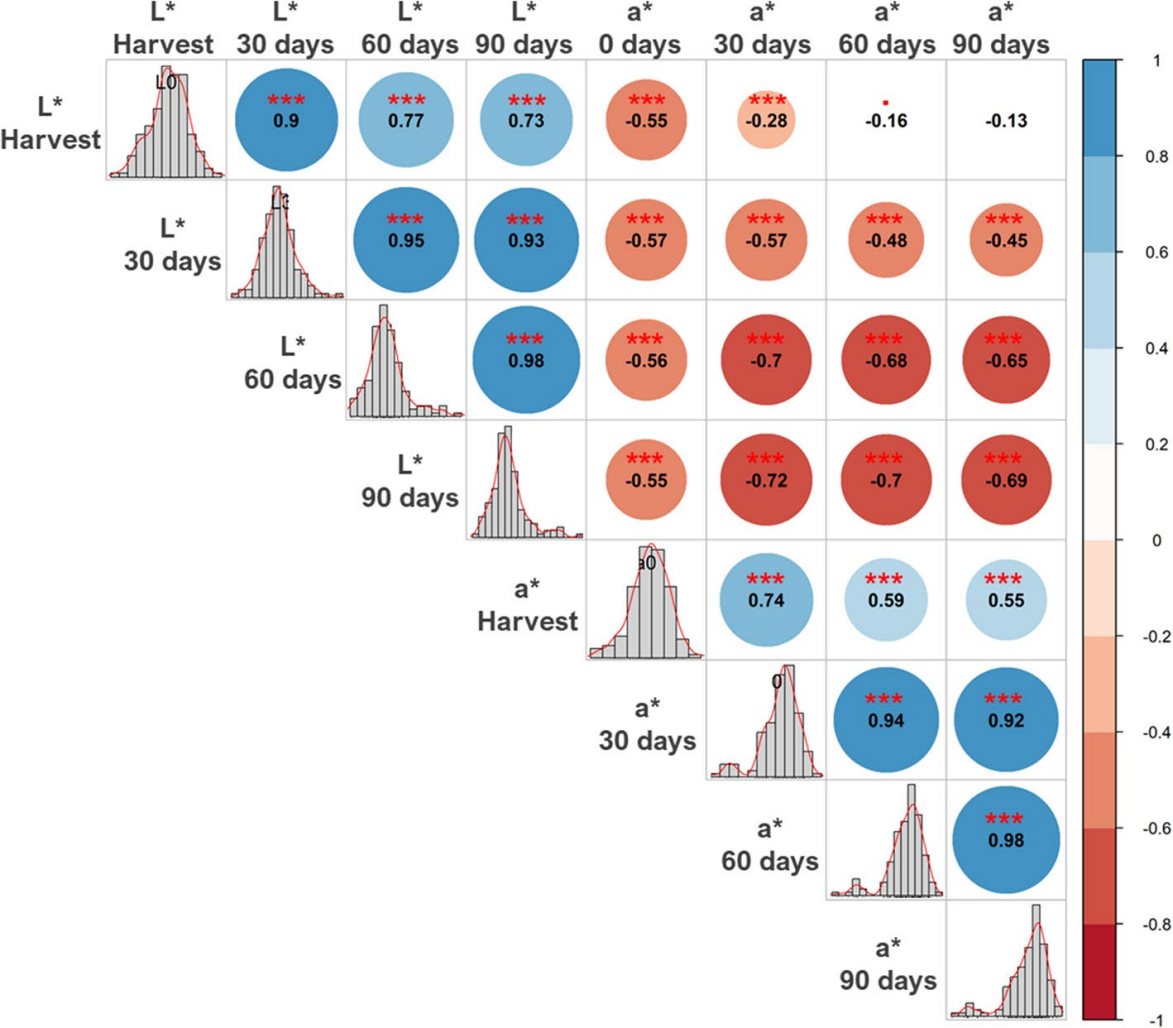
Pearson correlation analyses for the L* and a* color parameters at harvest time and at 30, 60, and 90 days after storage evaluated for the 138 carioca bean genotypes that composed the carioca diversity panel (CDP) in 2018

The phenotype data of the L* and a* parameters obtained from the three evaluation periods of the first experiment were used to select the shortest storage time with the highest correlation with the others. The Pearson correlation analyses showed significant correlations between all parameters and times, except between L* harvest vs a* 60 days and a* 90 days (Fig. 1). As expected, the correlation coefficients between the times of the same parameter were higher than between different ones. Although significant, the correlations between L* and a* were all negative, since higher values of L* and a* refer to lighter and less reddish samples, respectively.

In the case of the storage period, the time of 30 days was the shortest period with correlation greater than 90% for both parameters, and to reduce the days required for the second experiment, the 30-day storage time was selected. Considering the same storage times, the correlation values between L* and a* tend to increase with time, starting at 55% at harvest and reaching 69% after 90 days. However, the values showed that although the correlation was moderate to high, the two parameters did not have the same discriminatory power.

**Fig. 2:**
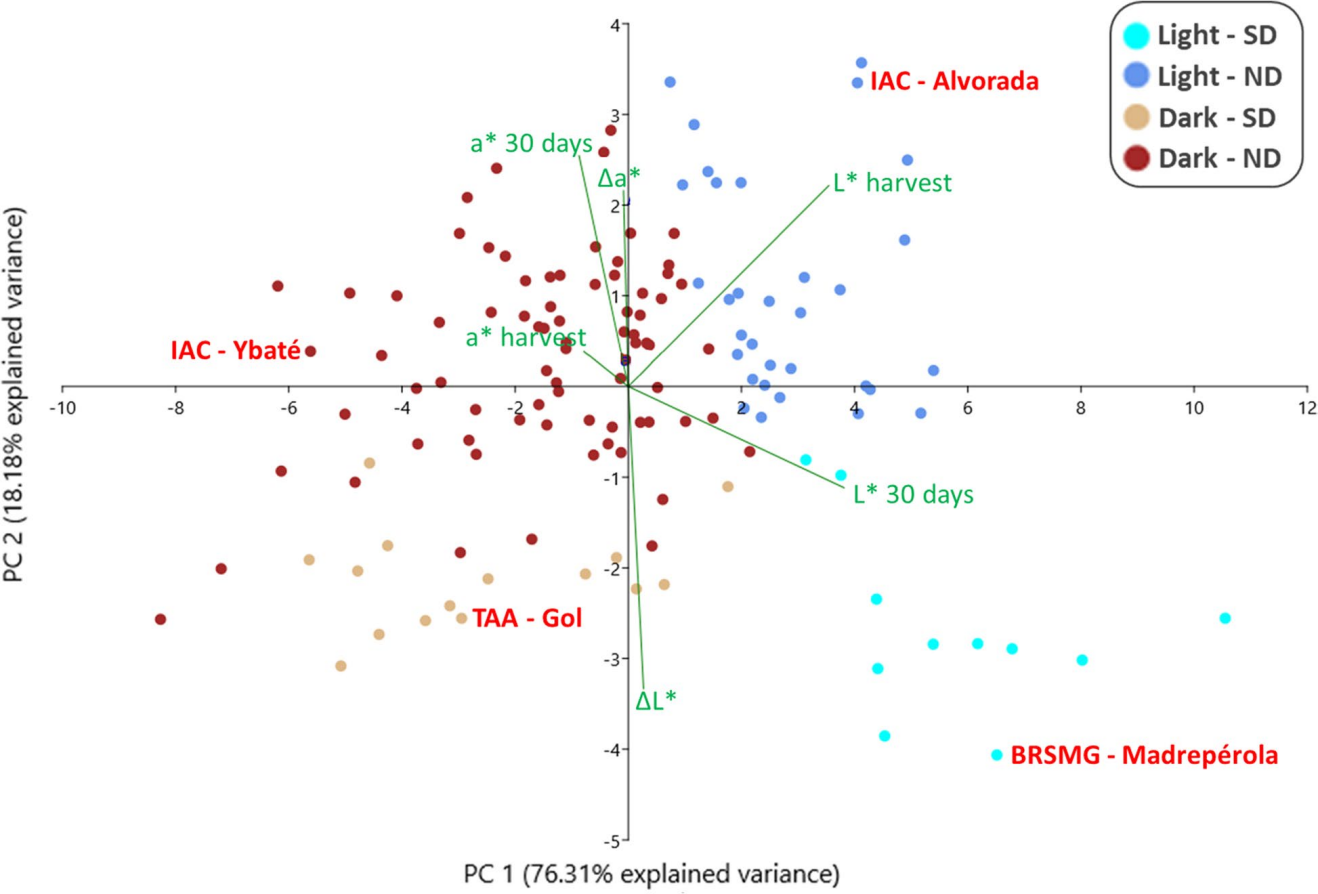
Principal component analysis biplot of L* and a* variables obtained from colorimeter readings of the 138 carioca genotypes at harvest time (L* harvest and a* harvest), after shelf storage of 30 days (L* 30 days and a* 30 days), and the delta measurements (ΔL* and Δa*). Dark blue dots, light blue dots, dark brown dots, and light brown dots represent the Light Regular Darkening, Light Slow Darkening, Dark Regular Darkening, and Dark Slow Darkening genotypes, respectively

Due to the moderate correlation between the L* and a* parameters, both were tested for discriminatory power by a PCA biplot (Fig. 2), aiming at selection of the best parameter for genetic mapping. The first component, explaining 76.3% of the observed variance, clearly separated the lightest from the darkest genotypes at harvest, while the second component separated the SD from the RD genotypes. Considering the vectors, the L* parameter exhibited a greater vector for the three variables (i.e., harvest, after storage, and delta) compared to the a* parameter, and because it explained a higher percentage of the phenotypic variation, this parameter was selected for the other analyses.

**Fig. 3:**
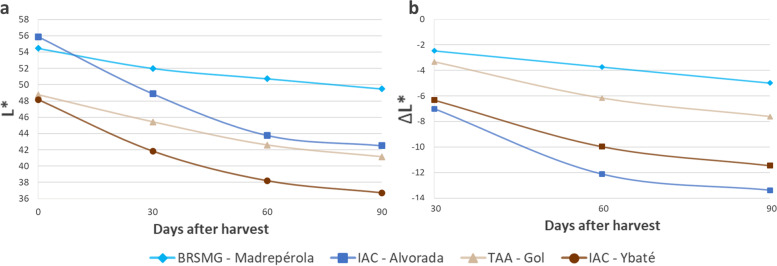
Plot for L* (**a**) and ΔL* (**b**) over time for BRSMG-Madrepérola, IAC-Alvorada, TAA-Gol, and IAC-Ybaté selected to represent the phenotypic classes of light SD, light RD, dark SD, and dark RD, respectively

The PCA biplot for L* (Fig. 2) took the standard cultivars for each phenotypic group (i.e., IAC-Alvorada [Light RD], BRSMG-Madrepérola [Light SD], IAC-Ybaté [Dark RD], and TAA-Gol [Dark SD]) to make the differences between the proposed groups even clearer (Fig. 3a). At harvest time, IAC-Alvorada and BRSMG-Madrepérola showed only 2.6% difference in lightness of seed coat color, and after 90 days of storage, this difference increased to 14% (Fig. 3a). However, comparing IAC-Alvorada with TAA-Gol, the difference at harvest time was 13%, and after storage, the difference dropped to only 3%. Thus, the results showed that considering only the SD and RD traits for genotype separation based on L* after storage does not provide adequate classification, since TAA-Gol would be classified as RD, but over time lost just -2.63 L* more than BRSMG-Madrepérola. Another important point when considering ΔL* (Fig. 3b) is that, unlike for L* (Fig. 3a) over time, for the ΔL* plot there was no change in the ranking of the genotypes (e.g., IAC-Alvorada had the highest L* at harvest but moved to second place after 30 days of storage) (Fig. 3b).

The average lightness value was 3% higher in the experiment performed in 2020 than in the experiment performed in 2018. However, considering the overall average, the genotypes darkened 39% more in the second experiment than in the first experiment (Table 1). When considering both experiments, the deviance analyses showed higher significance for the genotype effect and the genotype × environment interaction. In spite of that, the genetic correlation for the three traits was superior to 80%, indicating that although the environment influences the lightness of seed coat color of the genotype, the position of the genotype in the variation is not strongly affected (i.e., lighter-colored genotypes are always lighter colored). Moreover, the restricted variance interaction was low for all the traits evaluated, with the lowest being observed for ΔL*.

**Table 1:** Deviance analyses and the χ^2^ test, estimates of variance components, means, heritability, selective accuracy, coefficient of variation, and genotypic correlation among experiments for HL (Harvest L*), L30 (L* after 30 days) and ΔL* (Delta L*) traits evaluated in the 138 carioca bean genotypes from the carioca diversity panel (CDP) during the years 2018 and 2020

Effect	HL	L30	ΔL
Complete	1621.9	1665.2	1164.9
Genotype	141.8^a^	146.3^a^	75.8^a^
G × E	62.1^a^	70.1^a^	26.9^a^
Phenotypic variance	6.6	7.3	2.3
Genotypic variance	4.6	5.23	1.1
G × E variance	0.7	0.8	0.3
Residual variance	1.3	1.3	0.9
Mean experiment I	51.8 ± 2.35	46.8 ± 2.44	-4.9 ± 1.22
Mean experiment II	53.4 ± 2.81	45.2 ± 3.01	-8.2 ± 1.78
Overall mean	52.6 ± 2.71	46.0 ± 2.86	-6.6 ± 2.23
BRSMG-Madrepérola	54.4 ± 1.24	51.9 ± 1.22	-2.5 ± 1.46
IAC-Alvorada	55.9 ± 1.01	48.9 ± 1.47	-7.0 ± 2.25
TAA-Gol	48.8 ± 0.82	45.4 ± 1.00	-3.3 ± 1.52
IAC-Ybaté	48.2 ± 1.67	41.8 ± 0.61	-6.3 ± 1.99
Rgyear	0.87	0.87	0.81
h^2^	0.7 ± 0.08	0.7 ± 0.08	0.5 ± 0.07
Accuracy	0.94	0.95	0.89
CV%	2.15	2.49	14.47

HL: lightness (L*) of the seed coat at harvest; L30: lightness (L*) of the seed coat after 30 days of storage; ΔL: difference in final lightness (L30) and initial lightness (HL), h^2^: broad-sense heritability; Rgyear: genotypic correlation between years; CV: coefficient of variation

^a^ Significant at 1% by the *χ*^*2*^ test

As expected, the deviance analyses also showed high significance for the genotype effect, and the genetic variance of the traits L* Harvest (HL) and L* after 30 days (L30) represented more than 65% of the phenotypic variance, validating the use of the data for association mapping. In addition, the coefficient of variation of both traits was less than 2.5%, and the accuracy was greater than 90%. The delta L* (ΔL*) showed a moderate coefficient of variation value (14.5%), but the ΔL* depends on the HL and L30, which led to the largest variation observed. The same fact also explains the lower broad-sense heritability for the ΔL* trait, which was 0.5, while the others were 0.7.

### GWAS

**Fig. 4:**
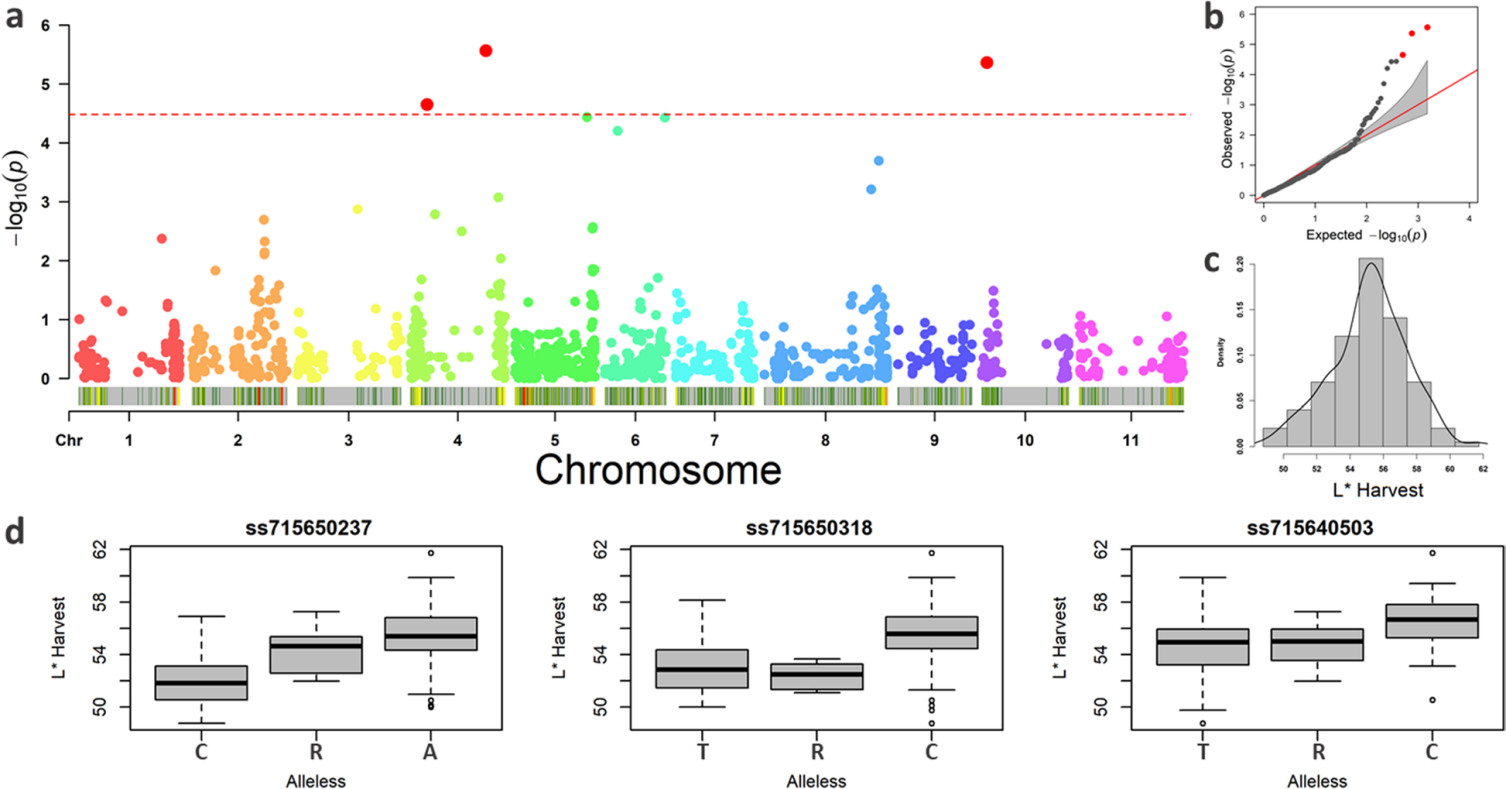
GWAS analyses using the carioca diversity panel (CDP) genotyped by 1,516 high-quality SNPs and evaluated for lightness (L*) at harvest with threshold defined by the Bonferroni test (cutoff α = 0.05). **a** Manhattan plots showing the association between the SNPs and the traits, **b** QQ-plot for the model fit, **c** histogram of phenotypic data, and **d** boxplots illustrating the relationships between alleles and phenotype for the significant SNPs

For association mapping, a total of 1,516 high-quality SNPs well distributed across the genome of the species were selected after *SNPCalling* using the 138 CDP genotypes. According to the Bonferroni test [30], the FarmCPU identified three significant QTL for HL (Fig. 4a), with a good fit of the model used (Fig. 4b) and with a normal distribution of phenotypic data (Fig. 4c). Two QTL were mapped on Pv04 (Table 2), one at the beginning of the chromosome associated with the SNP ss715650237 (position: 8.31 Mb) and the other at the end of the chromosome associated with the SNP ss715650318 (position: 38 Mb).

**Table 2:** GWAS results: significant SNPs for association mapping using the carioca diversity panel (CDP) genotyped by 1,516 SNPs, evaluating seed coat lightness at harvest (HL) and seed coat post-harvest darkening (L30 and ΔL*)

Trait	SNP	Chr	Position	*p* value	REF.a	ALT.a	MAF	Effect	nG
HL	ss715650237	Pv04	8,305,412	2.2E-05	C	A	0.09	-1.14	65
	ss715650318	Pv04	38,357,623	2.7E-06	T	C	0.12	-1.05	38
	ss715640503	Pv10	2,562,874	4.4E-06	T	C	0.27	0.66	88
L30	ss715646867	Pv01	42,399,057	5.3E-07	C	T	0.12	-1.02	100
	ss715648328	Pv04	5,499,065	5.1E-07	C	T	0.13	-1.11	83
	ss715650318	Pv04	38,357,623	1.6E-06	T	C	0.12	-0.94	38
	ss715647732	Pv07	32,272,598	1.8E-06	G	A	0.14	1.41	110
	ss715645851	Pv07	35,650,852	2.2E-05	A	G	0.08	-1.19	113
ΔL	ss715645575	Pv03	51,275,803	1.4E-05	C	T	0.21	0.37	118
	ss715646652	Pv08	58,966,240	7.8E-07	G	A	0.29	0.45	109
	ss715646104	Pv08	61,479,072	1.9E-06	A	G	0.24	-0.43	235
	ss715646121	Pv08	61,915,670	1.4E-05	C	T	0.15	-0.40	235
	ss715645744	Pv09	7,807,479	2.7E-06	C	T	0.32	-0.38	92
	ss715640510	Pv10	2,582,807	2.8E-05	C	T	0.26	-0.31	88

*Chr* Chromosomes, *Ref. a* Reference allele, *Alt.a* Alternative allele, *MAF* Minor allele frequency, *Effect* Allelic substitution effect, *nG* Number of genes in the 0.59 Mb confidence interval

The third QTL was identified at the beginning of the Pv10 at position 2.56 Mb and associated with the SNP ss715640503. Among the SNPs, both from Pv04 showed a greater effect; so, the alternative allele (i.e., 'A' allele for SNP ss715650237 and the 'C' allele for SNP ss715650318) contributed to greater genotype lightness (Fig. 4d). Two other QTL, one at the end of Pv05 and the other on Pv06, had a *p*-value very close to the significance limit – they would be considered significant at 6% significance. However, to avoid type I errors, neither were considered for further analyses.

**Fig. 5:**
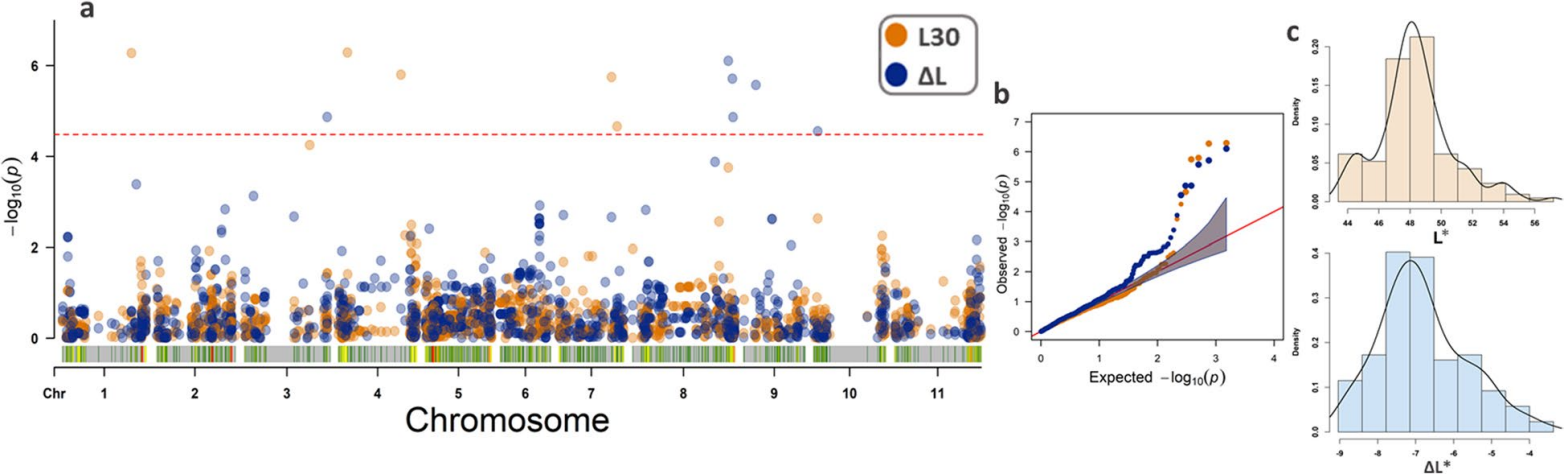
GWAS analyses using the carioca diversity panel (CDP) genotyped by 1,516 SNPs and evaluated for post-harvest seed coat darkening, with threshold defined by the Bonferroni test (cutoff α = 0.05). **a** Manhattan plots showing the association between the SNPs and the traits, where the orange color corresponds to the L* after 30 days of storage and the blue color to the ΔL* (final L* – initial L*); **b** QQ-plot for the model fit; and **c** histogram of phenotypic data

In order to identify QTL associated with tolerance to PHD, GWAS analyses were conducted with data from L30 and ΔL. Considering both traits, a total of 11 QTL were highly significant, though none were significant for both traits (Fig. 5a). The model used was a good fit for HL (Fig. 5b), and both phenotypic datasets also showed normal distribution (Fig. 5c).

In general, five QTL were significant for L30, two on each chromosome Pv04 and chromosome Pv07, and one on Pv01. The same SNP ss715650318 from Pv04 identified for HL showed significance for L30, and the second SNP ss715648328 on Pv04, also significant for L30, at 2.81 Mb from SNP ss715650237 was associated with HL. The SNPs on Pv07 were positioned at 3.38 Mb from each other, with the reference allele 'G' of SNP ss715647732 contributing to the greater lightness of the grain after storage, while the alternative 'G' allele of SNP ss715645851 was associated with lower lightness (Table 2).

Regarding the ΔL, six QTL showed high significance according to the Bonferroni test [30]. The first was associated with the SNP ss715645575 at position 51.28 Mb on Pv03. Three other QTL were identified on Pv08: the first was associated with SNP ss715646652 at position 58.97 Mb, and the other two were associated with SNPs ss715646104 and ss715646121, at 0.44 Mb from each other. They can be considered a single QTL between position 61.48 and 61.92 Mb. On chromosome Pv09, a single QTL was associated with SNP ss715645744 and identified at position 7.81 Mb. The last QTL associated with SNP ss715640510 was mapped on Pv10, at 0.02 Mb from SNP ss715640503, significant for HL.

### Candidate genes

Considering a confidence interval window of 0.59 Mb for each significant QTL, all SNPs were positioned close to a large number of genes (Table 2). The QTL with the smallest number of genes was associated with the SNP ss715650318 for HL and L30, with a total of 38 genes in the 1.18 Mb range. Both SNP ss715646104 and ss715646121 associated with ΔL* were positioned in the same QTL, the QTL with the largest number of genes, 235 total in a confidence interval of 1.62 Mb.

Among the candidate genes with a possible role in the traits evaluated, three copies of the gene coding ‘NAD(P)-binding rossmann-fold superfamily protein’ were identified at an average distance of 0.55 Mb from the SNP ss715650318 associated with HL and L30 (i.e., Phvul.004G112100, Phvul.004G112400, and Phvul.004G112501), one copy at 0.24 Mb from the SNP ss715646867 associated with L30 (i.e., Phvul.001G169500), and another copy at 0.35 Mb from the SNP ss715646652 associated with ΔL* (i.e., Phvul.008G237900). Genes encoding MYB transcription factors were also identified at 0.27 Mb from the SNP ss715650237 associated with HL (i.e., Phvul.004G057800), at 0.01 Mb from the SNP ss715648328 associated with L30 (i.e., Phvul.004G046000), and at 0.13 Mb from the SNP ss715645851 associated with L30 (i.e., Phvul.007G231800). The SNPs ss715646104 and ss715646652, associated with ΔL, also had copies of genes encoding the same protein from 0.55 and 0.49 Mb, respectively.

The Phvul.008G280400 gene encoding ‘oligopeptide-transporter’ and the Phvul.008G280700 gene encoding ‘sugar transporter protein’ were identified at 0.25 and 0.28 Mb from the SNP ss715646121 associated with ΔL, respectively. In addition, eight copies of genes encoding cytochrome P450 were identified in the SNP interval ss715640503 associated with HL. The same eight genes are in the SNP interval ss715640510 significant for ΔL, and another four encoding the same protein were identified for the same trait, one flanking the SNP ss715645575 and another three among the SNPs ss715646104 and ss715646121. For L30, a single copy was identified at 0.30 Mb from the SNP ss715645851 (i.e., Phvul.007G229700). All genes present in the confidence interval of each significant SNP for GWAS are given in Table S2.

## Discussion

Just as for darkening in pinto, cranberry, and red bean varieties, one of the most significant factors that lead to devaluation of carioca beans after harvest is seed coat darkening, because consumers presume, they are older and more difficult to cook [31]. However, carioca beans, unlike other varieties, are devalued not only through darkening over time, but those that have low seed coat lightness (i.e., darker beans) at harvest time are devalued for the producer for the same reason mentioned above [28, 32]. Therefore, Brazilian bean breeding programs have concentrated not only on developing carioca bean cultivars with tolerance to PHD, but those with the lightest colored grain possible [16], cultivars such as BRSMG-Madrepérola [33] and IAC-Polaco [34].

### Seed coat lightness at harvest

In regard to the carioca variety, the HL and the tolerance to PHD must be considered as different traits, and there is a certain misunderstanding in the literature. In this sense, the mapping of *loci* that control grain lightness at harvest and the identification of molecular markers associated with the trait are extremely important for breeding of carioca bean.

Studies have been carried out aiming to map the genetic control of tolerance to PHD [10, 21, 28, 31] and of grain color of the species [35–37]. Expression of seed coat color is controlled by a sequence of multiple alleles of the *P locus* that show allelic interactions with the *V locus* [38, 39], and they interact with alleles of seven other genes (*Gy*, *C*, *R*, *J*, *G*, B*,* and *Rk*) [35, 36]. Bassett [37] proposed a list of 24 genes that can affect the seed coat color trait, and, in the case of carioca beans, which are characterized by cream-colored seeds with brown stripes, the pattern of the stripes and the coloring is controlled by the *C locus* [40].

Our results show oligogenic inheritance for the HL trait, corroborated by the moderate heritability observed and the high genotypic correlation between the experiments. In addition, only three QTL were significant, two of which were mapped on Pv04. On the same chromosome, McClean et al. [36] identified the *G locus* linked to the OU14_900_ RADP marker, which was previously reported as controlling the yellow–brown factor in grain color [35]. The alignment of the forward sequence of the OU14_900_ against the reference genome, *Phaseolus vulgaris* v2.1 [5], showed that this marker is 7.26 Mb from the SNP ss715650318. Although the distance is considered large, the small mapping population (n80) used by McClean et al. [36] and the small number of markers may have led to low mapping resolution.

This is the first study conducted to identify QTL associated with HL in the carioca variety, and identification of the SNPs ss715650237, ss715650318, and ss715640502 has considerable potential for screening and selecting carioca common bean lines with lighter-colored grain in the early stages of the breeding process.

### Phenotypic tolerance to PHD

Previous studies classified the genotypes as SD and RD based only on evaluation of the color trait (i.e., diagrammatic scoring scale) or measurement of color (i.e., digital analysis and colorimeters) after a determined storage period, without considering the initial color of the grain [10, 16, 29, 31, 32]. Fundamentally, the SD trait should be associated with the fact that a given genotype loses little lightness of color over time, and not with the fact that it has light-colored grain after the storage period. Our results not only show that such classification is inadequate, as shown by the contrasting response of IAC-Alvorada and TAA-Gol (Fig. 3), but also that using the final evaluation without considering the initial color condition of the grain generates different results in identification of QTL associated with tolerance to PHD.

In addition to the SD and RD phenotypic classes, Elsadr et al. [22] proposed a third phenotypic class for pinto beans, the ND (non-darkening) class. Erfatpour et al. [12], using the same classification, mapped the *nd* QTL on chromosome Pv10 in a cranberry-like genotype. In the case of the carioca variety, among the 138 genotypes evaluated, all cultivars lost L* values greater than two points after 30 days, showing the absence of the ND phenotypic class for the variety. In addition, the cultivar considered as the standard for SD in previous studies [10, 23, 33, 41] was the second most tolerant genotype to PHD (-2.46 L*).

Several studies involving evaluation of carioca grain color used the L* parameter for breeding classification and selection [14, 16, 42]. Taking other bean varieties, such as pinto beans, into account, some studies have adopted the a* parameter [12, 21, 43]. Although there is no consensus in the literature, our results showed that for carioca bean, the L* parameter had greater discriminatory power. Regarding the time required for assessment of tolerance to PHD in the shelf storage method, some studies reported the need for 90 days [28, 42], while others evaluated up to six months after harvest [44].

Our results showed that the 30-day storage period is more than sufficient, as it showed correlation greater than 90% with the 90-day period, for both the L* and a* parameters. Silva et al. [16] and Silva et al. [10] also reported the 30-day storage period as best for selection of lighter-colored bean lines after storage, compared to the 60- and 90-day periods, mainly due to the high correlation indices and the shorter time required for evaluation.

### Genetic control of tolerance to PHD

The GWAS showed that the QTL involved in seed coat lightness after the storage period (L30) and in tolerance to PHD (ΔL*) are different, and it is noteworthy that only the L30 had significant QTL on the Pv07 chromosome close to the *sd locus* (i.e., SNP ss715647732 was positioned 2.5 Mb from the Pvsd-0028 marker) reported by Felicetti et al. [13] as associated with the SD trait in pinto beans. Rodrigues et al. [32] found that the same *locus* is responsible for tolerance to PHD in carioca beans, and Alvares et al. [23] validated the markers for selection of lighter-colored lines after storage. All the studies mentioned used only color after storage information, ignoring the initial color. In contrast, our results showed that the *sd locus* was not significant for tolerance to PHD (i.e., considering the ΔL* trait). Although the L30 trait can be associated with lighter or darker grains, the trait does not express change in color (i.e., change in lightness over storage).

Considering the ΔL* to be the trait that most efficiently expresses tolerance to PHD, six QTL were mapped on four different chromosomes. This is the first study involving GWAS for tolerance to PHD, and unlike previous studies that reported monogenic control for the trait [16, 21, 22, 32], our results showed oligogenic control. Corroborating the more complex profile of the trait, broad-sense heritability was the lowest estimated (i.e., 0.5) and the genotype × environment interaction was highly significant. Silva et al. [16], Silva et al. [10], Siqueira et al. [15], and Alvares et al. [28] also reported genotype × environment interaction for the trait, corroborating the oligogenic profile.

The same QTL positioned at the beginning of the Pv10 chromosome associated with HL was significant for ΔL, showing that although that QTL is not the one with the greatest effect, it is associated with two traits of extreme importance for the variety. Erfatpour et al. [12] also mapped a QTL associated with the non-darkening trait on Pv10; however, the QTL reported by the authors for the cranberry-like bean genotype cannot be considered the same QTL associated with the carioca variety, due to the distance of the *loci* from each other. Regarding the QTL found on Pv03, there are no previous studies reporting a *locus* mapped on this chromosome. Nevertheless, the large number of genes for the SNP ss715645575 confidence interval emphasizes the need for future studies for identification of candidate genes.

Both QTL identified on the Pv08 are at 2.51 Mb, the second QTL being associated with two significant SNPs in a range of 0.02 Mb. The proximity of the three significant SNPs and both QTL shows the possibility of a single and a large *locus* associated with the trait. Couto et al. [29] were the first to identify molecular markers linked to QTL for tolerance to PHD in carioca beans and reported the PVESTBR-98 marker flanking the only one mapped QTL. The BLASTN of the forward sequence of the marker against the reference genome (i.e., *Phaseolus vulgaris* v2.1, [5]) showed that it was aligned 0.06 Mb from the SNP ss715646104 in the second QTL that we identified on Pv08. In addition, McClean et al. [36] mapped the *C locus* associated with the pattern of the seed coat at the end of the same chromosome. The authors also identified the *T locus* associated with the pattern of the seed coat linked to the marker OM19400 on chromosome Pv09. The BLASTN of the forward sequence of the marker OM19400 showed that it is at 3.87 Mb from the SNP ss715645744.

Our results showed that possibly the* C* and *T loci* reported in control of the pattern of the seed coat may be associated with tolerance to PHD in carioca beans. Future studies are needed to achieve a better understanding of the influence of both *loci* on control of light-coloredness in carioca beans over time.

### Candidate genes for breeding

The PHD trait depends on a series of chemical processes. In the case of the cranberry bean variety, studies have shown that the total phenolic content was significantly higher in RD than in ND genotypes [45, 46]. Myers et al. [47] reported genes encoding the ‘NAD(P)-binding rossmann-fold superfamily protein’ associated with the total phenolic content, given its involvement in the biosynthesis of flavonoids. In this regard, the Phvul.008G237900 gene identified in the interval of the first QTL on Pv08 associated with ΔL* has considerable potential for studies of differential expression, since it has the same annotation as the genes reported by Myers et al. [47].

Among the various groups of polyphenolic compounds, flavonoids are the most common, such as flavonols, anthocyanins, and proanthocyanidins [12]. According to Freixas-Coutin et al. [25], the precursors of the flavonoid biosynthesis pathway are catalyzed by the enzymes Cytochrome P450 (CYTOCHROME P450), flavonoid 3'-hydroxylase, and flavonoid 3′5'-hydroxylase. On Pv10, we identified seven copies of genes encoding the Cytochrome P450 enzyme for the QTL associated with both HL and ΔL, making these genes (Phvul.010G013100, Phvul.010G013000, Phvul.010G012900, Phvul.010G012700, Phvul.010G019100, Phvul.010G019600, and Phvul.010G022400) potential candidates for genetic breeding.

In addition, the MYB-like genes identified for both HL and ΔL* also show great potential since the phenylpropanoid pathway genes are thought to be regulated by MYB-bHLH-WDR complexes [48, 49]. MYB proteins are key factors in regulatory networks controlling development metabolism, including the synthesis of anthocyanins [50]. Erfatpour et al. [12] also reported MYB-like genes as one of the main candidates when identifying genes associated with tolerance to PHD.

Finally, enzymes that regulate the transport of molecules such as ‘oligopeptide transporter’ and ‘sugar transporter’ may also play a crucial role in the accumulation of proanthocyanidin, given that the greater expression of these genes is correlated with lighter-colored grain in all stages of the cranberry bean variety, as indicated by Freixas-Coutin et al. [25]. Therefore, the Phvul.008G280400 and Phvul.008G280700 genes identified in the SNP ss715646121 confidence interval significant for ΔL* are also potential candidates for improving this trait.

## Conclusions

This is the first associative mapping study for the lightness of seed coat color at harvest and tolerance to PHD, both traits of extreme importance for the main commercial varieties of common beans. Our results showed qualitative control for the HL trait in carioca beans, and three QTLs showed high significance, enabling the identification of candidate markers for use in assisted selection and germplasm screening strategies. Regarding the evaluation of tolerance to PHD, our phenotypic results showed that the parameter L * has greater discriminatory power and that 30 day of storage is strategically the best period for PHD evaluation. Regarding the separation into phenotypic classes, our results showed the presence of four distinct phenotypic classes, and that for the correct assessment of tolerance to PHD, it is necessary to consider the initial and final seed coat color (ΔL*). Considering the ΔL*, we identified six significant QTLs on four different chromosomes, with the Pv10 QTL being the most interesting since it was associated with both HL and PHD traits. In addition, the *sd locus* reported in previous studies, showed significance for the carioca diversity panel used only considering the L* parameter after the storage period, and therefore, not representing the tolerance to PHD.

## Materials and Methods

### Plant material, genotyping, and SNPCalling

For the present study, the carioca diversity panel (CDP) was used, composed of 138 carioca accessions selected from the germplasm bank of the Common Bean Breeding Group at Agronomic Institute (IAC) to represent the genetic diversity of the Brazilian carioca bean. The accessions were genotyped by high-throughput genotyping technologies using the Illumina BeadChip BARCBean6K_3 [51]. The CDP was validated for GWAS by Almeida et al. [9] and consists of the main commercial cultivars of carioca bean in Brazil, from the first cultivar (i.e., ‘Carioca comum’) released in 1971 [52] to more modern cultivars, such as IAC-1850, released in 2019 [53]. The set also includes genotypes with contrasting seed coat darkening rates identified in previous studies, with the RD cultivars BRS-Cometa, BRS-Estilo, BRS-Pérola, BRS-Pontal, BRS-Requinte, IAC-Alvorada, and BRS-Majestoso [10, 16, 17, 23, 28, 29, 32] and the SD cultivars BRS-Madrepérola, ANFC-9, Branquinho, and TAA-Dama [28, 32, 33].

Quality analysis of the genotypic data was performed using the TASSEL 5.0 software [54], eliminating SNPs with minor allele frequency < 3%, heterozygosity > 5%, and missing data > 10%. The high-quality genotypic matrix was converted into HAPMAP file format, with the reference allele represented by “A”, the alternative allele by “G”, the heterozygous alleles by “R”, and missing data by “N”. The BARCBean6K_3 was developed based on the first common bean genome (i.e., *Phaseolus vulgaris* v1), and therefore, the flanking sequences of each SNP were blasted (i.e., BLASTN) against the most current reference genome, *Phaseolus vulgaris* v2.1 [5], and the position of each SNP was obtained. Markers with unknown position in the genome were removed, and the imputation of “N” markers was performed using the Beagle 5.0 software [55]. All the information regarding the CDP is given in Table S1, including the phenotypic and genotypic data.

### Evaluation of HL and seed coat PHD

The experiments were carried out at the Fazenda Santa Eliza experimental station (IAC, Campinas, SP, Brazil) in two different years, with the first experiment sown in August 2018 and the second in July 2020. The experimental plot was composed of 1-m rows with 10 plants and 0.5-m spacing between rows, following a completely randomized block experimental design with three replications. Harvest was performed manually, and the pods remained in a greenhouse for 10 days for total drying and color standardization of the grain. The grain was sieved and stored in paper bags kept in a dark and dry storage room at 10 ± 2 °C.

A random sample of approximately 80 seeds from each plot, enough to fill a 6-cm Petri dish, was used for analysis with a colorimeter (Konica Minolta CR-410, Osaka, Japan), which was expressed by the mean of five technical repetition measurements for each sample. The color parameters were determined with the colorimeter according to the CIELAB system [56], where three axes were used to describe color: the L* parameter (i.e., luminosity) on the z-axis, which is a darkness (i.e., the value of L* = 0 being perfectly black) to lightness (i.e., the value of L* = 100 being perfectly white) component; the a* parameter on the x-axis, which is a greenness (i.e., the value of a* = -60 being perfectly green) to redness (i.e., the value of a* =  + 60 being perfectly red) component; and the b* parameter on z-axis, which is a blueness (i.e., the value of b* = -60 being perfectly blue) to yellowness (i.e., the value of b* =  + 60 being perfectly yellow) component. The L* a* b* color system works in a homologous way to the human eye, in addition to being a uniform color scale, enabling comparison of color values between different samples [57].

After color measurement at harvest, the samples were packed in zip-lock bags (8.5 × 12 cm, 80 g each) and stored on shelves under controlled temperature (26 ± 2 °C) and photoperiod (12 h.) conditions in a room containing fluorescent lamps. Every week, the samples were repositioned, aiming at homogeneity of light exposure on the samples. For the first experiment, the color of each plot was measured at 30, 60, and 90 days of storage, aiming at selection of the best time for evaluation of the second experiment, for which the time of 30 days of storage was adopted for darkening assessment.

### Phenotypic statistical analyses

Over time, the bean seed coat tends to become darker and reddish, so only the L* and a* parameters were considered for the initial analyses. In order to determine the shortest storage time that would represent the total time adopted, the Pearson correlation values (r^2^) of the L* and a* parameters in the four reading times were estimated from the phenotypic data of the first experiment using the R package Corrplot [58]. The storage time with an r^2^ value greater than 90% compared to the other reading times, for both the L* and a* parameters, was adopted for color analysis of the second experiment.

**Fig. 6:**
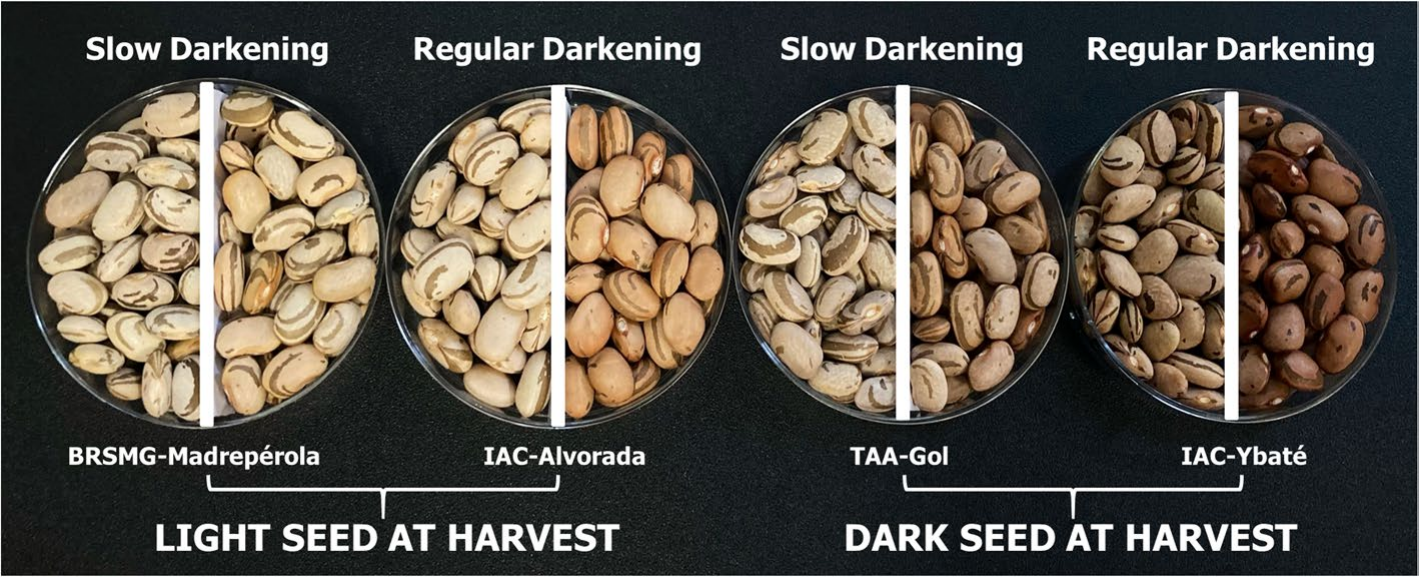
Example of phenotypic division into four contrasting classes, the first division being in relation to the luminosity (L*) of the seed at harvest (light × dark) and the second, in relation to tolerance to post-harvest seed coat darkening (slow × regular darkening). The genotypes MRSMG-Madrepérola, IAC-Alvorada, TAA-Gol, and IAC-Ybaté were selected to represent the phenotypic classes of Light Slow Darkening, Light Regular Darkening, Dark Slow Darkening, and Dark Regular Darkening, respectively

For selection of the parameter that would best explain the phenotypic variation, the colorimeter data at harvest and after 30 days of storage, as well as the delta of both parameters (i.e., ΔL* = final L*—initial L*; Δa* = final a*—initial a*), were used for the biplot of the principal component analysis (PCA) through the PAST4.05 program [59]. The parameter with the largest vectors for the three variables (i.e., harvest, after 30 days, and delta) was selected for analysis of association mapping and estimates of genetic parameters. For best visualization of the data, the genotypes were divided into 4 phenotypic classes (Fig. 6):

- **Light SD:** genotypes with light-colored seeds at harvest (L* ≥ 53) with slow darkening (ΔL* ≥ -4), with BRSMG-Madrepérola as the standard cultivar.
- **Light RD:** genotypes with light-colored seeds at harvest (L* ≥ 53) with regular darkening (ΔL* ≤ -4.1), with IAC-Alvorada as the standard cultivar.
- **Dark SD:** genotypes with dark-colored seeds at harvest (L* ≤ 52.9) with slow darkening (ΔL* ≥ -4), with TAA-Gol as the standard cultivar.
- **Dark RD:** genotypes with dark-colored seeds at harvest (L* ≤ 53.9) with regular darkening (ΔL* ≤ -4.1), with IAC-Ybaté as the standard cultivar.

In order to evaluate the genotype × environment interaction and to validate the phenotypic data for GWAS, the deviance analysis (ANADEV), broad-sense heritability, and variance components were estimated by Restricted Maximum Likelihood/Best Linear Unbiased Predictor (REML/BLUP) by the SELEGEN software [60].

### Genomic association model

The fixed and random model circulating probability unification—FarmCPU [61] implemented in the rMVP R package [62] was used for association mapping due to its high statistical power and greater sensitivity to QTL with lesser effects. The package explores the multi-*locus* mixed model and performs the analysis in two interactive steps: a fixed-effect model is applied first, followed by a random-effect model. Both models were repeated interactively until no significant marker was detected. To avoid type I errors (i.e., false positives), the structuring matrix was tested using the Bayesian Information Criterion (BIC) test according to Schwarz [63], for a regular mixed linear model [64] with the first five components of the principal component analysis (PCA, Table 3S Supplementary material). According to Schwarz [63], the highest BIC reveals the best number of covariates for the model. As shown by Almeida et al. [9], the CDP does not require the use of a structuring matrix to correct type I errors (i.e., false positives), since there are no subgroups in the set. The *p-*value threshold of each SNP in the model was determined by the Bonferroni [30] threshold method (cutoff α = 0.05). The phenotypic matrix used was given by the genotypic values estimated by the Restricted Maximum Likelihood / Best Linear Unbiased Estimator (REML / BLUE) using the lmerTest R package [65].

### Candidate genes and genetic annotation

The physical position of all the significant SNPs was used for the thorough search for candidate genes through genetic annotation, which was inferred using the *Jbrowse* from the Phytozome v11.0 [66] and the reference genome *Phaseolus vulgaris* v2.1 [5]. For the search, a confidence interval window of 0.59 Mb was considered, the average distance identified by Almeida et al. [9] for the CDP (i.e., distance to LD decay = r^2^ 0.2). LD decay was estimated using squared allele-frequency correlation intrachromosomal pairs, with the R package LDcorSV [67], accounting for the relatedness (Kinship [68]). The LD decay curves for all the chromosomes (Figure S1, Supplementary material) was explained using the nonlinear model proposed by Hill and Weir [69], as described by Diniz et al. [70].

## Supplementary Information

**Additional file 1: Figure S1**. (a) Principal component analysis based on the 1, 516 SNPs for the carioca diversity panel (n = 138); (b) Kinship heatmap plot showing the population relationship, estimated according to VanRaden [68]. (c) Linkage disequilibrium (LD) decay determined by the LD measurements (r2) against the distance between SNPs (Mb) for the 11 chromosomes (Pv) adjusted according to the model proposed by Hill and Weir [69] controlled for relatedness.

**Additional file 2: Table S1.** Cultivar name, grain size (mm), commercial classification, institution of origin, genealogy, adjusted phenotypic mean (BLUE) for lightness at harvest and tolerance to PHD, and genotypic matrix for the 138 carioca common bean genotypes of the carioca diversity panel (CDP).

**Additional file 3: Table S2.** Genes positioned within the confidence interval of each significant SNP for association mapping of the HL, L30, and ΔL* traits, as well as the genetic annotation of each gene identified.

**Additional file 4: Table 3S.** Bayesian Information Criterion (BIC) test according to Schwarz [63], for all traits evaluated using the first five components of the principal component analysis.

## Data Availability

All data generated or analysed during this study are included in this published article.
