# Supplementary figures and images for: Genome-wide association mapping reveals new loci associated with light-colored seed coat at harvest and slow darkening in carioca beans
Source: BMC Plant Biol. 2021 Jul 20;21:343. doi: 10.1186/s12870-021-03122-2 (PMC8290572; doi:10.1186/s12870-021-03122-2)

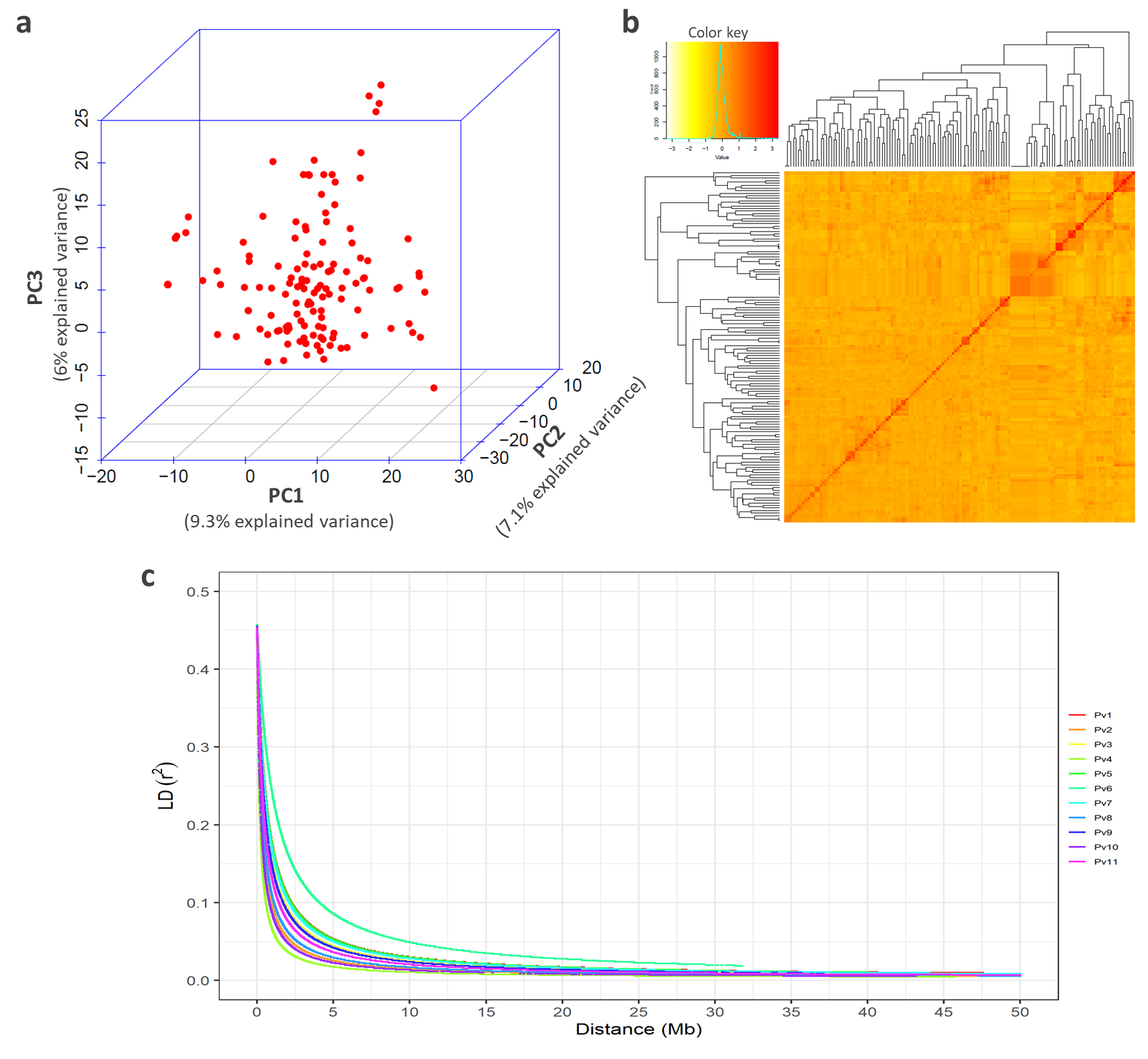

Supplement: Supplementary file 1 — Additional file 1: Figure S1. (a) Principal component analysis based on the 1, 516 SNPs for the carioca diversity panel (n = 138); (b) Kinship heatmap plot showing the population relationship, estimated according to VanRaden [68]. (c) Linkage disequilibrium (LD) decay determined by the LD measurements (r2) against the distance between SNPs (Mb) for the 11 chromosomes (Pv) adjusted according to the model proposed by Hill and Weir [69] controlled for relatedness. [file 12870_2021_3122_MOESM1_ESM.tif]
